# (How) Can We Reduce Violence Against Women by 50% over the Next 30 Years?

**DOI:** 10.1371/journal.pmed.1001761

**Published:** 2014-11-25

**Authors:** Rachel Jewkes

**Affiliations:** Gender and Health Research Unit, Medical Research Council, Pretoria, South Africa

## Abstract

In this month's editorial, *PLOS Medicine* Editorial Board member Rachel Jewkes identifies challenges that the violence prevention research community faces in substantially reducing violence against women and girls over the next 30 years.

*Please see later in the article for the Editors' Summary*

Each year, interpersonal violence is experienced and perpetrated by millions of people worldwide. In 2010, it was the 27th cause of death globally, causing an estimated 456,268 deaths worldwide [1]. Violence against women has been shown to be highly prevalent globally, with partner violence affecting one in three women, and one in 15 women (7%) having been raped by a man who was not a partner [2],[3]. Recognising this huge global burden, the 67th World Health Assembly adopted the resolution “Strengthening the Role of the Health System in Addressing Violence, in Particular against Women and Girls, and against Children” [4] and mandated countries globally to develop violence prevention through their health sector. The goal of reducing violence by 50% over the next 30 years has been mooted by the World Health Organization as a rallying point for the global violence prevention community and was the subject of critical debate at the recent Global Violence Reduction Conference 2014 at King's College, Cambridge University, UK, which was hosted by the Institute of Criminology Violence Research Centre and the World Health Organization [5]. Whilst ostensibly ambitious, several high-income countries, including the United States, have reduced rates of some forms of violence by 50% or more over a very short period of time, and such reductions are supported by historical trends of reduced homicide over several centuries in several European countries [6]–[8]. There is no real evidence, however, that violence against women is reducing in low- and middle-income countries [3]. Indeed, in South Africa, where there has been considerable gender activism and growth in women's empowerment, non-fatal rape and intimate partner violence seem quite resistant to change, notwithstanding the measured reductions in female homicide [9],[10]. The key question, then, is how can we secure substantial reductions in violence against women in low- and middle-income countries?

## Reducing Fragmentation of Research Efforts

One challenge for the violence research community is to build a stronger knowledge base comprised of research from across multiple research disciplines and relevant to different forms of violence. Currently, knowledge production is largely siloed, with researchers focused on specific forms of violence, yet there are enormous overlaps between forms of violence. For example, knowledge sharing between the fields of youth violence prevention and gender-based violence prevention is limited, and yet rape is an important form of youth violence [11],[12]. The same was true, until fairly recently, between the child abuse prevention and gender-based violence fields. Here there are increasing moves—for example, by the Sexual Violence Research Initiative and donors such as the UK Department for International Development—to address the two problems together, recognising that childhood adversity is an important risk factor for violence against women, as well as the conceptual difficulties of imposing a distinction between rape of younger and older females [13],[14].

Different research disciplines have, over the years, brought different strengths and insights to the field of violence prevention, and have their own limitations. Inter-disciplinary research is needed to build on the strengths of, and avoid conceptual stunting by boundaries of thought within, particular research disciplines. For example, standard epidemiological approaches to research have often served to accentuate differences (e.g., in types of violence and types of risk factors and behaviour) through the reduction of violence, factors, and behaviour into measurable units for surveys, and have rarely encouraged avenues of analysis that deepen understanding of latent (unmeasurable) constructs, such as constructions of masculinity and femininity. One of the most important advances in understanding violence causation has been at the nexus of sociology and epidemiology, through the application of gender theory—in particular, understanding patterns of risk factors stemming from underlying groups of behaviours that map onto constructions of masculinity [12],[15],[16].

## Prioritising Evidence-Based Approaches

Stakeholders must give more recognition to the science of preventing violence against women. Gender-based violence has been an underdeveloped and underfunded field, notwithstanding the systematic development and remarkable innovation of some violence prevention interventions [17]–[19]. Recent reviews of evidence have highlighted that gender-based violence prevention interventions and strategies are often developed ad hoc, with limited opportunity to learn from intervention success or failure. Furthermore, adoption of interventions in different settings often has more to do with effective marketing than with scientific evidence [20]. Building evidence-based interventions and prevention strategies requires theory to be applied at multiple levels [15], including understanding risk factors and understanding the theory of the problem to be tackled, for example, understanding in a broad theoretical sense how gender inequity or social norms operate. Application of theory also requires an understanding of what drives and enables change, for example, how approaches that view social settings or institutions ecologically (i.e., as systems that have social norms that influence behaviour, as well as individual factors and policies/laws and institutional practices) may be necessary to secure change in them [21]. It also requires an understanding of methods that are effective for achieving changes (e.g., awareness raising or behaviour change) in specific populations and settings. There is a need to develop packages of interventions or intervention strategies that can be applied at scale. However, a balance needs to be struck so that local innovation and acknowledgment of complexity and differences between contexts can be accommodated.

Where there are more rigorous evaluations, the shortage of funds has resulted in a literature that is characterised by under-powered studies, and heated debate over whether interventions would have “worked” if they had been larger [22],[23]. There is no recognisable product pipeline, and only a handful of organisations globally have ever produced (or adapted) more than one theoretically grounded intervention to reduce violence against women. It's hard to see how these problems can be addressed without large and long-term funding for centres of excellence in gender-based violence intervention development—centres that develop and improve multiple interventions, coordinate testing and trials so that evaluations are comparable, and focus on development of appropriate human resources.

## Funding a Violence Prevention Pipeline

There is a pressing need to change the global architecture of the violence prevention field to foster the science of gender-based violence prevention, and there are important lessons to learn here from fields like drug discovery. The limited funding for violence prevention has been largely responsible for the fragmentation of the field, and to date, there has been limited support from very large donors. The Department for International Development's £25 million research and innovation fund “What Works to Prevent Violence against Women and Girls” is the first large investment to systematically build the field. Substantial advances in intervention development require proper investment and deployment of highly trained, creative minds. Perhaps there is a need to replicate the product pipeline seen in, for example, microbicide development, with some form of equivalent of scientifically based progress from early stage concepts, to pre-clinical research, to phase 1–3 trials. The microbicide development field has been coordinated by the Alliance for Microbicide Development in a way that is completely unparalleled in violence prevention, and this coordination has been essential for the speed and scale of R&D that has been undertaken [24]. A strong prevention knowledge platform requires large donors to come to the table in violence prevention and provide sustainable funding for the architecture of the field, so that physical or virtual centres of excellence can be established, research can be coordinated, and the skills, knowledge, and experience required can be nurtured and sustained.

Changes in the violence prevention field needed to reach ambitious targets for reducing violence against women require a much deeper understanding across policy makers and donors of the preventability of violence. The evidence of change from the United States and other countries in this regard is crucial [6],[7]. We have to build recognition that reducing rates of violence globally by 50% within 30 years is not just something that must happen, but it's something that *can* happen. Achieving this requires significant changes to the gender-based violence prevention field, and a new era of large-scale funding and coordination.

## References

[1] Institute for Health Metrics and Evaluation (2013) GBD heat map. Seattle (Washington): Institute for Health Metrics and Evaluation.

[2] Abrahams N, Devries K, Watts C, Pallitto C, Petzold M, Shamu S, et al (2014) Worldwide prevalence of non-partner sexual violence: a systematic review. Lancet 383: 1648–1654. 24529867 10.1016/S0140-6736(13)62243-6

[3] Devries KM, Mak JY, Garcia-Moreno C, Petzold M, Child JC, et al (2013) Global health. The global prevalence of intimate partner violence against women. Science 340: 1527–1528. 23788730 10.1126/science.1240937

[4] th World Health Asembly (2014) Strengthening the role of the health system in addressing violence, in particular against women and girls, and against children. WHA67.15. Available: http://apps.who.int/gb/ebwha/pdf_files/WHA67/A67_R15-en.pdf?ua=1. Accessed 23 October 2014.

[5] Butchart A, Mikton C (2014) World Health Organisation—welcome. In: University of Cambridge Institute of Criminology Violence Research Centre, World Health Organisation. Global Violence Reduction Conference 2014: strategies to reduce violence by 50% in the next 30 years—programme. p. 3. Available: http://www.vrc.crim.cam.ac.uk/conference/cbooklet/cbookletPDF. Accessed 24 October 2014.

[6] Eisner M (2011) Human evolution, history and violence: an introduction. Br J Criminol 51: 473–478.

[7] Finkelhor D, Jones L (2006) Why have child maltreatment and child victimisation declined? J Soc Issues 62: 685–716.

[8] Eisner M (2014) From swords to words: does macro-level change in self-control predict long-term variation in levels of homicide? In: Tonry M, editor. Why crime rates fall and why they don't. Chicago: University of Chicago Press.

[9] Machisa M, Jewkes R, Lowe-Morna C, Rama K (2011) The war at home: Gender Based Violence Indicators Project. Gauteng Research Report. Johannesburg: Gender Links and South African Medical Research Council.

[10] Abrahams N, Mathews S, Martin LJ, Lombard C, Jewkes R (2013) Intimate partner femicide in South Africa in 1999 and 2009. PLoS Med 10: e1001412. 23565064 10.1371/journal.pmed.1001412 PMC3614499

[11] Jewkes R, Sikweyiya Y, Morrell R, Dunkle K (2011) Gender inequitable masculinity and sexual entitlement in rape perpetration South Africa: findings of a cross-sectional study. PLoS ONE 6: e29590. 22216324 10.1371/journal.pone.0029590 PMC3247272

[12] Fulu E, Warner X, Miedema S, Jewkes R, Roselli T, et al. (2013) Why do some men use violence against women and how can we prevent it? Quantitative findings from the United Nations Multi-country Study on Men and Violence in Asia and the Pacific. Bangkok: United Nations Development Programme, United Nations Population Fund, UN Women, United Nations Volunteers.

[13] Knerr W, Gardner F, Cluver L (2011) Parenting and the prevention of child maltreatment in low- and middle-income countries: a systematic review of interventions and a discussion of prevention of the risks of future violent behaviour among boys. Pretoria: Sexual Violence Research Initiative.

[14] Jewkes R (2012) Rape perpetration: a review. Pretoria: Sexual Violence Research Initiative.

[15] Jewkes R, Flood M, Lang L (2014) From working with men and boys to changing social norms and reducing inequities in gender relations: a paradigm shift in prevention of violence against women and girls. Lancet In press. 10.1016/S0140-6736(14)61683-4 25467578

[16] Fulu E, Jewkes R, Roselli T, Garcia-Moreno C (2013) Prevalence of and factors associated with male perpetration of intimate partner violence: findings from the UN Multi-country Cross-sectional Study on Men and Violence in Asia and the Pacific. Lancet Glob Health 1: e187–e207. 25104345 10.1016/S2214-109X(13)70074-3

[17] Welbourn A (1995) Stepping stones. Oxford: Strategies for Hope.

[18] Abramsky T, Devries K, Kiss L, Francisco L, Nakuti J, et al (2012) A community mobilisation intervention to prevent violence against women and reduce HIV/AIDS risk in Kampala, Uganda (the SASA! Study): study protocol for a cluster randomised controlled trial. Trials 13: 96. 22747846 10.1186/1745-6215-13-96 PMC3503643

[19] Katz J, Heisterkamp AH, Fleming W (2011) The social justice roots of the mentors in violence prevention model and its application in a high school setting. Violence Against Women 17: 684–686. 21628336 10.1177/1077801211409725

[20] Fulu E, Kerr-Wilson A, Lang J (2014) What works to prevent violence against women and girls? Evidence review of interventions to prevent violence against women and girls. Pretoria: Medical Research Council.

[21] Morrell R, Epstein D, Unterhalter E, Bhana D, Moletsane R (2009) Towards gender equality? South African schools during the HIV/AIDS epidemic. Pietermaritzburg: University of KwaZulu-Natal Press.

[22] Hossain M, Zimmerman C, Kiss L, Abramsky T, Kone D, et al (2014) Working with men to prevent intimate partner violence in a conflict-affected setting: a pilot cluster randomized controlled trial in rural Cote d'Ivoire. BMC Public Health 14: 339. 24716478 10.1186/1471-2458-14-339 PMC4007144

[23] Abramsky T, Devries K, Kiss L, Nakuti J, Kyegombe N, Starmann E, et al (2014) Findings from the SASA! Study: a cluster randomized controlled trial to assess the impact of a community mobilization intervention to prevent violence against women and reduce HIV risk in Kampala, Uganda. BMC Med 12: 122. 25248996 10.1186/s12916-014-0122-5 PMC4243194

[24] Alliance for Microbicide Development (2007) Mapping the microbicide effort. Silver Spring (Maryland): Alliance for Microbicide Development.

